# circFLNA promotes intestinal injury during abdominal sepsis through Fas-mediated apoptosis pathway by sponging miR-766-3p

**DOI:** 10.1007/s00011-023-01688-1

**Published:** 2023-01-10

**Authors:** Ling Ye, Yuan Shi, Huifeng Zhang, Chao Chen, Jingjing Niu, Jianxu Yang, Zhifeng Li, Huanzhang Shao, Bingyu Qin

**Affiliations:** Department of Critical Care Medicine, Henan Key Laboratory for Critical Care Medicine, Zhengzhou Key Laboratory for Critical Care Medicine, Henan Provincial People’s Hospital, Zhengzhou University People’s Hospital, Henan University People’s Hospital, Zhengzhou, 450003 Henan China

**Keywords:** circFLNA, Intestinal injury, Abdominal sepsis, Fas, Apoptosis

## Abstract

**Background:**

Intra-abdominal infections are the second most common cause of sepsis in the intensive care unit. Intestinal epithelial injury due to abdominal sepsis results in a variety of pathological changes, such as intestinal bacteria and toxins entering the blood, leading to persistent systemic inflammation and multiple organ dysfunction. The increased apoptosis of intestinal epithelial cells induced by sepsis further exacerbates the progression of sepsis. Although several studies have revealed that circRNAs are involved in intestinal epithelial injury in sepsis, few studies have identified the roles of circRNAs in intestinal epithelial apoptosis.

**Methods:**

We used laser capture microdissection to obtain purified epithelial cells located in intestinal crypts from four patients with abdominal sepsis induced by intestinal perforation and four samples from age and sex-matched non-septic patients. Microarray analysis of circRNAs was conducted to assess differentially expressed circRNAs between patients with and without sepsis. Lastly, in vitro and in vivo assays were performed to study the mechanism of circFLNA in intestinal epithelial apoptosis during sepsis.

**Results:**

circFLNA was upregulated in the intestinal epithelium after abdominal sepsis induced by intestinal perforation. Inhibition of miR-766-3p impaired si-circFLNA-mediated inhibition of apoptosis and inflammation factor levels in lipopolysaccharide (LPS)-treated HIEC-6 cells. circFLNA aggravated apoptosis and inflammation through the Fas-mediated apoptosis pathway in both LPS-treated HIEC-6 cells and a mouse cecal ligation and puncture model.

**Conclusion:**

Our findings showed that circFLNA promotes intestinal injury in abdominal sepsis through the Fas-mediated apoptosis pathway by sponging miR-766-3p. The circFLNA/miR-766-3p/Fas axis has potential as a novel therapeutic target for treating intestinal injury in sepsis.

**Supplementary Information:**

The online version contains supplementary material available at 10.1007/s00011-023-01688-1.

## Introduction

Intra-abdominal infections are second only to pneumonia as the most common cause of sepsis in the intensive care unit (ICU) patients, with a high incidence of mortality [1, 2]. Once severe abdominal sepsis or septic shock is identified, the mortality rate can be as high as 42.3% [3, 4]. Despite the implementation of optimal management principles, such as early diagnosis, personalized fluid resuscitation, damage control, empiric antibiotic therapy, and organ support in the ICU, the mortality due to abdominal sepsis has not significantly improved over the past decades [5, 6]. Based on a previous study, the increase in intestinal permeability caused by mucosal injury in sepsis results in a variety of pathological changes such as intestinal bacteria and toxins entering the blood, leading to persistent systemic inflammation and multiple organ dysfunction [7, 8]. It has been reported that apoptosis of intestinal epithelial cells (IECs) is an important biological process which leads to intestinal injury [9]. Nevertheless, the detailed mechanisms that regulate IEC apoptosis in abdominal sepsis are still not fully elucidated.

Circular RNAs (circRNAs), a novel class of non-coding RNAs with covalently closed loop structures, are widely found in eukaryotic cells and exhibit tissue-specific expression [10]. The sequence of circRNAs is highly conserved among different species and is resistant to ribonuclease R (RNase R) because of their circular structure [11]. Instead of coproducts of RNA splicing, circRNAs mainly function as sponges of microRNAs (miRNAs) and regulate the expression of target genes [12]. miRNAs repress gene expression by binding to complementary sequences in the 3ʹ untranslated region (3ʹ-UTR) of target mRNAs [13]. Recent studies have shown that circRNAs are aberrantly expressed in sepsis and have the potential to serve as diagnostic biomarkers and therapeutic targets [14, 15]. For instance, circHIPK3 serves as a sponge for miR-29b to enhance the repair of intestinal epithelial cells in patients with sepsis [16]. Furthermore, circTLK1 promotes inflammation and oxidative stress via the miR-106a-5p/HMGB1 axis in sepsis-associated acute kidney injury, and hsa_circ_0003091 mediates sepsis-induced lung injury by reducing miR-149 [17, 18]. Although several studies have revealed that circRNAs are involved in intestinal epithelial injury in sepsis [16, 19, 20], few studies have investigated the roles of circRNAs in intestinal epithelial apoptosis.

Laser capture microdissection (LCM) is a microscopic-guided system that can isolate specific cell types or regions of interest from tissue sections with a laser beam [21]. A variety of studies have indicated that LCM is powerful for isolating distinct cells and is helpful for the subsequent analysis of DNA, RNA, or protein profiles by sequencing or microarray [22–24]. With respect to the intestine, LCM has been used to obtain high-integrity RNA samples from freshly resected human intestinal tissue and to evaluate the gene expression profiles between crypts and villi of ileal epithelial cells [25, 26]. Furthermore, compared to villi, intestinal crypts have been shown to contribute to antimicrobial activity by expressing certain proteins [27]. In addition, intestinal stem cells (ISCs), which renovate the intestinal epithelium, are located at the bottom of crypts [28]. Therefore, we used LCM to obtain particular cells from the intestinal crypts of patients with abdominal sepsis induced by intestinal perforation and non-sepsis patients separately and then analyzed the circRNA expression profiles using circRNA microarray.

In this study, we found that circFLNA was upregulated in the intestinal crypts of patients with intestinal perforation. Moreover, we discovered that circFLNA originated from exons 9–15 of the *FLNA* gene, and its expression was significantly increased in HIEC-6 cells after being treated with lipopolysaccharide (LPS). Further mechanistic studies revealed that circFLNA promotes intestinal epithelial apoptosis and inflammation via the miR-766-3p/Fas axis in vitro and in vivo.

## Materials and methods

### Patients intestinal tissues

The resected intestinal tissues were obtained from 20 patients with intestinal perforation diagnosed with sepsis according to Sepsis 3.0. Discarded tissues resected from 23 patients with gastrointestinal stromal tumors were used as controls. All patients provided informed consent, and this study was approved by the Ethics Committee of Henan Provincial People’s Hospital.

### Laser capture microdissection and CircRNA microarray analysis

Highly purified epithelial cells located in intestinal crypts from four patients with abdominal sepsis induced by intestinal perforation and four samples from age- and sex-matched non-sepsis patients were obtained with LCM, according to a previously described protocol [29]. The enriched circRNAs were sent to BoHao Bio-tech (Shanghai, China) for circRNA microarray analysis after the total RNA was digested with ribonuclease R (RNase R).

### Cell culture and chemicals

Normal HIEC-6 cells, human undifferentiated crypt enterocytes, were acquired from the Type Culture Collection of the Chinese Academy of Sciences (Shanghai, China). The cells were incubated in Dulbecco’s modified Eagle’s medium (DMEM, Gibco; Grand Island, NY, USA) supplemented with 10% fetal bovine serum (FBS, Gibco) and cultured in a humid atmosphere at 37 °C with 5% CO_2_ in air. LPS from *Escherichia coli* O111:B4 was purchased from Sigma-Aldrich (St Louis, MO, USA).

### Total RNA extraction, actinomycin D assay, RNase treatment, and quantitative real-time PCR (qRT-PCR)

Total RNA, including circRNA, was extracted from intestinal tissues and HIEC-6 cells using RNAiso Plus (TaKaRa, Shiga, Japan) according to the manufacturer’s instructions. The transcription of RNAs extracted from HIEC-6 cells was blocked with 2 μg/mL actinomycin D (Sigma-Aldrich) for 8, 16, and 24 h. The RNAs extracted from HIEC-6 cells were treated with RNase R (Abcam, Cambridge, UK) for 30 min at 37 ℃ according to the protocol previously described [30]. Nuclear and cytoplasmic RNAs were isolated using the Cytoplasmic & Nuclear RNA Purification Kit (Norgen Biotek, Ontario, Canada), according to the manufacturer’s instructions. qRT-PCR was performed using a SYBR-Green PCR Master Mix Kit (Takara) on a QuantStudio Dx system (Applied Biosystems, CA, USA). The abundance of circRNAs and mRNAs was normalized to that of GAPDH, and small nuclear U6 was used as an endogenous control for miRNAs. The relative expression of circRNAs, miRNAs, and mRNA was measured using the 2^−ΔΔCt^ (Ct; cycle threshold) method. The primers used for qRT-PCR are listed in Supplementary Table S1.

### Fluorescence in situ hybridization and immunofluorescent staining

The signals of Cy5-labeled probes specific to circFLNA were detected using a fluorescence in situ hybridization (FISH) kit (GenePharma, Shanghai, China) according to the manufacturer’s instructions. Nuclei were counterstained with 4’, 6-diamidino-2-phenylindole (DAPI). Immunofluorescence (IF) staining analysis to detect Fas localization in HIEC-6 cells was performed according to a previously described protocol [31]. Images were acquired using a TCS SP2 AOBS confocal microscope (Leica Microsystems, Mannheim, Germany).

### Plasmid construction and transfection

Lentiviral vectors encoding circFLNA, small interfering RNAs (siRNA) targeting circFLNA, miRNA-766-3p mimics, and miRNA-766-3p inhibitor sequences were designed and synthesized by BioLink (Shanghai, China). The Fas (CD95) gene was synthesized according to the mRNA sequence of the human Fas gene and cloned into a lentiviral vector. HIEC-6 cells were transfected with 50 nM overexpressing circFLNA (circFLNA OE), si-circFLNA, Fas, miR-766-3p mimics, miR-766-3p inhibitors, or the corresponding controls using Lipofectamine RNAiMax (Invitrogen, Waltham, MA, USA) according to the manufacturer’s protocol.

### Cell viability and apoptosis assay

Cell viability was measured using the Cell Counting kit-8 (CCK8) (Dojindo, Kumamoto, Japan), according to the manufacturer’s protocol. HIEC-6 cells were treated with LPS at concentrations of 0, 1, 5, 10, 20, and 50 μg/mL for 24 h, and the optimal concentration of LPS was obtained. HIEC-6 cells transfected with lentivirus circFLNA OE, si-circFLNA, or the corresponding control were treated with 50 μg/mL LPS. Absorbance at 450 nm was measured using a Gen5 microplate reader (BioTek, Vermont, USA). Cell apoptosis was detected according to the protocol described in our previous study [32]. A BD FACSCalibur Flow Cytometer (BD Biosciences, CA, USA) was used to evaluate the apoptosis rate according to the manufacturer’s instructions.

### Enzyme-linked immunosorbent assay (ELISA)

Cytokines in the supernatants of HIEC-6 cells and mouse intestinal homogenates were measured using ELISA kits (R&D Systems, Minneapolis, MN, USA), according to the manufacturer’s protocol. The mouse intestinal tissues were homogenized using a homogenizer, and the supernatant was collected after centrifugation at 800 × *g* for 20 min at 4 °C. The concentrations of interleukin (IL)-6, IL-1β, and tumor necrosis factor alpha (TNF-α) were determined using ELISA kits (R&D Systems). The optical density (OD) at 450 nm was measured using a Gen5 microplate reader (BioTek). A standard curve was plotted before sample concentrations were calculated. The experiments were repeated in triplicate.

### Protein microarray analysis

The protein lysate samples of LPS-treated HIEC-6 cells were analyzed using the Human Cytokine Antibody Array 4000 (RayBio, GA, USA), according to the manufacturer’s instructions. Briefly, the array slide was incubated with 65 µL of LPS-treated HIEC-6 cell lysate overnight at 4 °C and equilibrated to room temperature on the following day. Then, the array slide was incubated for 2 h after extensive washing with an array-specific biotinylated antibody cocktail. The slide was incubated with Cy3-equivalent dye-conjugated streptavidin for 1 h. Finally, an InnoScan 300 microarray scanner (Innopsys, IL, USA) was used to obtain the images. To minimize false-positive hits, each sample was screened twice, and only the hits that appeared on both screenings were analyzed.

### Western blot analysis

Western blot analysis was performed according to the protocol in our previous study [32]. The following antibodies were purchased as indicated: anti-Fas, anti-FADD, anti-Caspase-8, anti-Caspase-3, anti-occludin, anti-ZO-1 (Cell Signaling Technology, MA, USA), and anti-β-actin (Sigma-Aldrich).

### Bioinformatic analysis

The target miRNAs interacting with circFLNA were predicted using the publicly available databases circInteractome (https://circinteractome.nia.nih.gov/index.html) and circBank (http://www.circbank.cn/). The miRNAs targeting Fas were predicted using TargetScan (https://www.targetscan.org/vert_80/). The miRNAs presented in all three databases were validated in HIEC-6 cells.

### Dual-luciferase reporter assay

HIEC-6 cells were transfected with either wild-type (WT) or mutant (MUT) circFLNA vector, miR-766-3p mimics, or mimics normal control (NC), and WT or MUT Fas 3′-UTR reporter using Lipofectamine 2000 (Invitrogen). The dual-luciferase reporter assays (Promega, Madison, WI, USA) were performed according to the manufacturer’s instructions. After transfection for 48 h, a GloMax fluorescence reader (Promega) was used to assess luciferase activity.

### RNA immunoprecipitation

The Magna RIP RNA-Binding Protein Immunoprecipitation Kit (Millipore, Bedford, MA, USA) was used to perform the RNA Immunoprecipitation (RIP) assays according to the manufacturer’s instructions. Briefly, HIEC-6 cells were transfected with miR-766-3p mimics or mimics NC and then cells were lysed using RIPA buffer (Beyotime Biotechnology, China) after 48 h. Magnetic beads were incubated with anti-AGO2 and anti-rabbit IgG as controls (Cell Signaling Technology) for 30 min. The cell lysates were immunoprecipitated with coated magnetic beads on a table concentrator at 4 °C overnight. The following day, RNAiso Plus was used to isolate the co-precipitated RNA bound to the beads. The expression of circFLNA and miR-766-3p in the isolated RNAs was detected using qRT-PCR.

### Animals and cecal ligation and puncture (CLP) model

C57BL/6 N mice (8–10 weeks old and 20–25 g) were obtained from the Experimental Animal Center of Zhengzhou University. The mice were maintained in a temperature-controlled room (22 °C ± 2 °C) with free access to water and food. Animal experiments were performed according to the principles of the Declaration of Helsinki and were approved by the Institutional Animal Ethical Committee of Zhengzhou University.

The CLP model of abdominal sepsis was established as previously described [33]. In brief, the abdomen was opened by making a tiny incision (1 cm) in the middle of the abdomen after anesthetization with pentobarbital sodium (50 mg/kg), and the cecum was exposed. The cecum was ligated in the middle and perforated twice using a 20-gauge needle. Subsequently, a small amount of feces was squeezed out of the perforation. The abdominal incision was stitched layer-by-layer before returning the cecum to the peritoneal cavity. For mice in the sham group, the abdomen was opened to expose cecum, but no ligation or perforation was performed. When the procedure was complete, saline (50 mL/kg) was subcutaneously injected for resuscitation.

### Animal grouping and specimen collection

In this study, the mice were divided into six groups (*n* = 25), and the treatment for each group was as follows. The sham group (1 mL saline was injected via tail vein 24 h before sham surgery), CLP group (1 mL saline was injected via tail vein 24 h before CLP surgery), CLP + si-NC + inhibitor-NC group (10 μg si-NC and 10 μg inhibitor-NC were injected via tail vein 24 h before CLP surgery), CLP + si-NC + miR-766-3p inhibitor group (10 μg si-NC and 10 μg miR-766-3p inhibitor were injected via tail vein 24 h before CLP surgery), CLP + si-circFLNA + inhibitor-NC group (10 μg si-circFLNA and 10 μg inhibitor-NC were injected via the tail vein 24 h before CLP surgery), and CLP + si-circFLNA + miR-766-3p inhibitor group (10 μg si-circFLNA and 10 μg miR-766-3p inhibitor were injected via the tail vein 24 h before CLP surgery). After surgery, 15 mice in each group were randomly allotted to analyze the survival rate over 7 days, and the remaining 10 mice in each group were used to collect blood and intestinal tissues at 48 h. Blood was collected from the hearts of mice using a micro-injector, and serum was obtained after centrifugation. Intestinal tissues were harvested after anesthetization by intraperitoneal injection of pentobarbital sodium. Part of intestinal tissues was instantly frozen in liquid nitrogen and stored at − 80 °C to detect inflammatory factors and extract proteins. The remaining tissues were fixed in 10% formalin to conduct the subsequent histological experiments.

### Detection of intestinal mucosal permeability

The levels of d-lactic acid in the serum were detected using a d-lactic acid assay kit (Megazyme, Wicklow, Ireland) according to the manufacturer’s instructions. The levels of diamine oxidase (DAO) in the serum were measured using an ELISA kit (R&D Systems) according to the manufacturer’s instructions. Absorbance at an excitation wavelength of 450 nm was measured using a Gen5 microplate reader (BioTek). FD-40 (750 mg/kg) was administered by gavage to the mice in each group 18 h after surgery. Venous blood samples were collected from the mesentery 6 h later, and serum was obtained after centrifugation. The absorbance at an excitation wavelength of 450 nm and an emission wavelength of 520 nm was measured using a Gen5 microplate reader (BioTek). These experiments were repeated in triplicate.

### Hematoxylin and eosin staining, immunohistochemistry, and TdT-mediated dUTP-biotin nick end-labeling staining

After fixation in 10% formalin, the intestinal tissues were embedded in paraffin wax and cut into 3 μm slices. The slices were then dehydrated with gradient alcohol, cleaned with xylene, and sealed with resin. Hematoxylin and eosin (H&E) staining was performed on the slices. Finally, images were captured using a light microscope (Nikon, Tokyo, Japan) to detect histopathological changes. The severity of intestinal injury was assessed according to Chiu’s scoring system, as previously described [9].

For immunohistochemistry (IHC), tissue slices were treated with anti-Fas antibody (1:500 dilution; Cell Signaling Technology). The horseradish peroxidase (HRP)-conjugated secondary antibody (Gene Tech, Shanghai, China) was incubated for 30 min at room temperature and used to detect the primary antibody. The images were acquired using a light microscope (Nikon), and Fas staining was quantified using Image-Pro Plus 7 (Media Cybernetics, MD, USA).

Apoptotic cells in the intestinal epithelium sections were detected using TdT-mediated dUTP-biotin nick end-labeling (TUNEL) reagent (Elabscience, Wuhan, China) according to the manufacturer’s instructions. Images were acquired using a fluorescence microscope (Leica Microsystems), and TUNEL-positive cells were quantified using Image-Pro Plus 7 (Media Cybernetics).

### Statistical analysis

Statistical analyses were performed using the SPSS statistical software program version 22 (IBM, IL, USA). Two-tailed Student’s *t*-test was applied to compare the differences between two groups, and one-way analysis of variance (ANOVA) was used to compare differences among multiple groups. Survival analysis was performed using the Kaplan–Meier method, and differences between the survival curves were assessed using log-rank tests. Statistical significance was set at *P* < 0.05.

## Results

### Circular RNA expression profiles in intestinal epithelium of individuals with sepsis

**Fig. 1:**
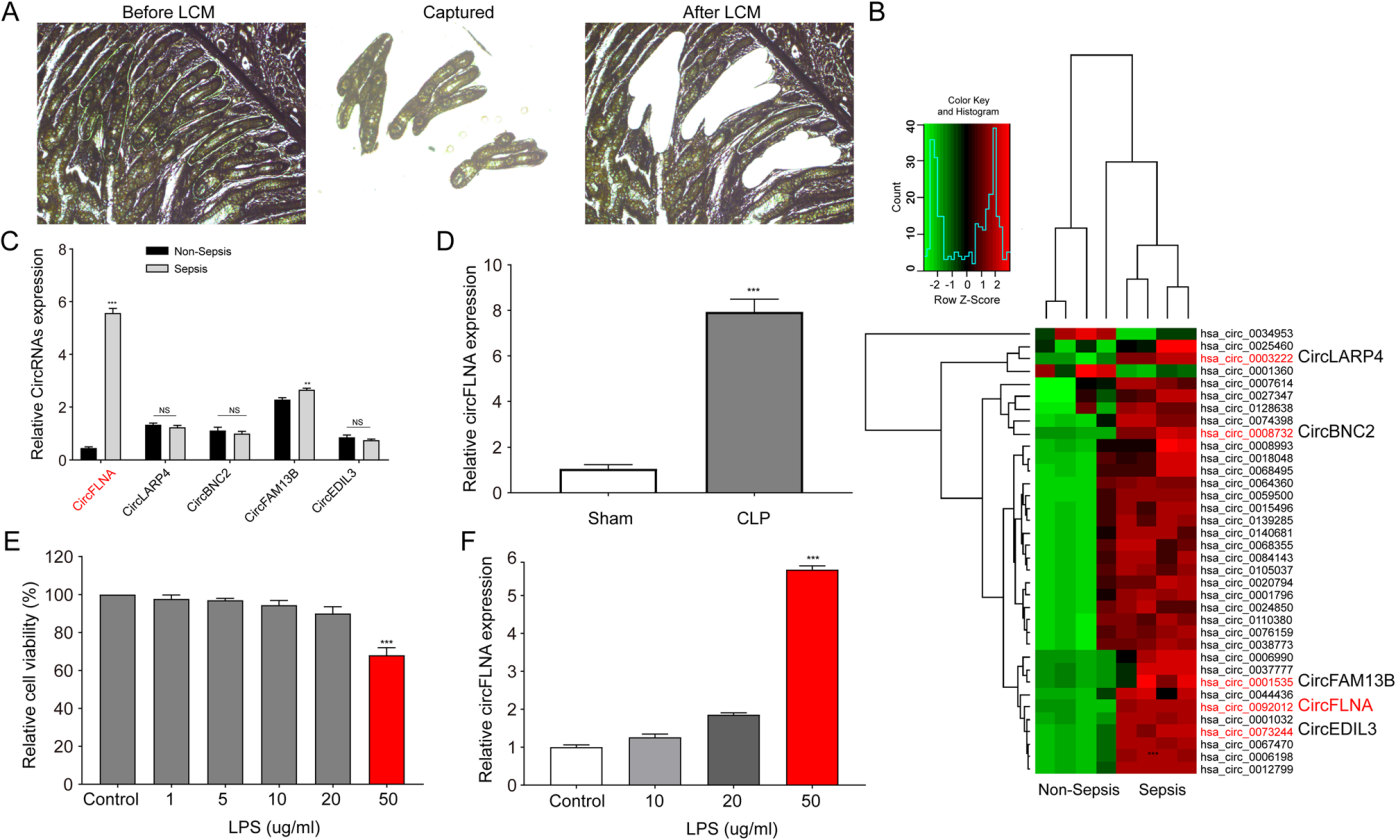
circRNA expression profiles in intestinal epithelium from sepsis sample. **A** Intestinal epithelial cells of patients with or without sepsis were obtained from fixed tissue sections using laser capture microdissection. **B** Heat map of differentially expressed circRNAs between septic and non-septic human intestinal epithelial cells. **C** Validation of circFLNA and other differentially expressed circRNAs by qRT-PCR in the intestinal mucosa from 20 patients with sepsis and 23 non-sepsis patients. **D** circFLNA was upregulated in the intestinal mucosa of mice exposed to CLP for 48 h. **E** HIEC-6 cells were treated with increasing concentrations of LPS for 24 h and cell viability assays were performed using a CCK-8 kit. **F** circFLNA levels in HIEC-6 cells treated with different concentrations of LPS for 24 h

Homeostasis disorder of the intestinal epithelium plays an important role in sepsis pathogenesis. Highly purified intestinal epithelial cells located in intestinal crypts from four patients with intestinal perforation and four samples from age- and sex-matched patients without sepsis were captured using LCM (Fig. 1A). To assess the expression profile of circRNAs isolated from purified intestinal epithelial cells, microarray analysis of circRNAs was conducted. Total RNAs were treated with RNase R to digest linear RNAs. Unsupervised hierarchical clustering showed differentially expressed circRNAs (fold-change (FC) > 2 or < 0.5, *P* < 0.05) between septic and non-septic intestinal epithelial cells, including 34 upregulated circRNAs and 2 downregulated circRNAs (Fig. 1B). Table 1 shows the most upregulated 12 circRNAs (FC > 2.5, *P* < 0.05) in sepsis tissues compared with their expression in non-sepsis tissues. We selected five highly expressed circRNAs (circFLNA, circLARP4, circBNC2, circFAM13B, and circEDIL3) with a high FC > 3.0 (*P* < 0.001) for further validation.

**Table 1:** Differential circRNAs expression in purified intestinal epithelial cells between four abdominal sepsis and four non-sepsis patients

circRNA	*P* value	Fold change	Regulation	CircRNA type	Gene symbol
hsa_circ_0001535	< 0.001	6.23	Up	Exonic	*FAM13B*
hsa_circ_0092012	< 0.001	5.85	Up	Exonic	*FLNA*
hsa_circ_0073244	< 0.001	4.36	Up	Exonic	*EDIL3*
hsa_circ_0003222	< 0.001	3.79	Up	Exonic	*LARP4*
hsa_circ_0008732	< 0.001	3.13	Up	Exonic	*BNC2*
hsa_circ_0006990	0.03	2.92	Up	Exonic	*VAPA*
hsa_circ_0037777	0.02	2.84	Up	Exonic	*ALG1*
hsa_circ_0044436	0.005	2.82	Up	Exonic	*KAT7*
hsa_circ_0001032	0.04	2.73	Up	Exonic	*TET3*
hsa_circ_0067470	0.008	2.63	Up	Exonic	*STAG1*
hsa_circ_0006198	0.006	2.59	Up	Exonic	*LCOR*
hsa_circ_0012799	0.002	2.53	Up	Exonic	*DOCK7*

The intestinal mucosae obtained from 20 patients with sepsis and twenty-three non-sepsis patients were used to verify the expression of these circRNAs. As shown in Fig. 1C, the expression of circFLNA and circFAM13B was increased in patients with sepsis compared with that in non-septic patients, while the expression of circBNC2 and circEDIL3 was not significantly different between the two groups. Among the two significantly upregulated circRNAs in patients with sepsis, circFLNA expression was upregulated. Furthermore, we also detected the expression of circFLNA in the mouse CLP model and found that circFLNA expression was increased in the intestinal mucosa of the CLP groups compared with that in the sham group (Fig. 1D). Thus, circFLNA was selected for in vitro validation. First, HIEC-6 cells were treated with different concentrations of LPS to construct an in vitro sepsis model. The viability of HIEC-6 cells decreased after treatment with increasing LPS concentrations for 24 h (Fig. 1E). The cell viability was obviously reduced at an LPS concentration of 50 µg/mL and the falling range reached almost 40%. Moreover, qRT-PCR analysis revealed that circFLNA expression was increased more significantly at an LPS concentration of 50 µg/mL than at 10 and 20 µg/mL (Fig. 1F). The concentration of LPS used to treat HIEC-6 cells for further experiments was selected as 50 µg/mL.

### CircFLNA is overexpressed in LPS-treated HIEC-6 cells and mainly localized in the cytoplasm

**Fig. 2:**
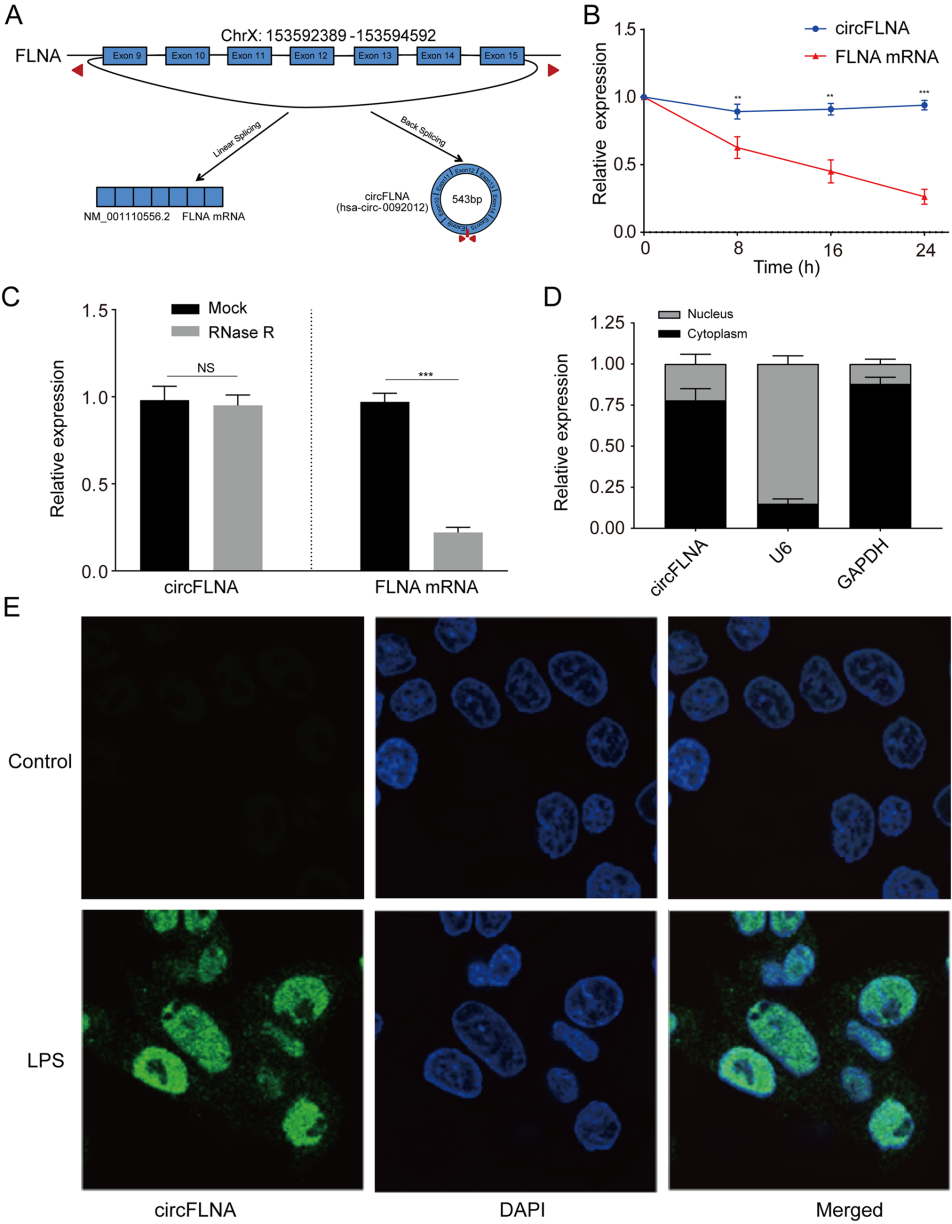
circFLNA is overexpressed in LPS-treated HIEC-6 cells and mainly localized in the cytoplasm. **A** A schematic illustration shows that exon 9–15 derived from FLNA formed circFLNA. **B** qRT-PCR assays were performed to detect the expression of circFLNA and linear FLNA mRNA in HIEC-6 cells treated with actinomycin D at a specific time point. **C** After total RNA treatment with or without RNase R, the expression of circFLNA and linear FLNA mRNA in HIEC-6 cells was detected using qRT-PCR. **D** A nuclear-cytoplasmic fractionation assay revealed that circFLNA was mainly detected in the cytoplasm. **E** RNA-FISH assay showed that circFLNA was predominantly localized in the cytoplasm of HIEC-6 cells after treatment with LPS for 24 h. Nuclei stained blue with DAPI. Values are shown as mean ± standard deviation. *NS* not significant. *P < 0.05, **P < 0.01, and ***P < 0.001

circFLNA, with a spliced mature sequence of 543 bp in length, was generated by reverse splicing of exons 9–15 of the *FLNA* gene, located at chrX:153592389–153594592 (Fig. 2A). Convergent primers were designed to amplify the linear FLNA mRNA, and divergent primers were designed to amplify circFLNA. To determine the stability of circFLNA, HIEC-6 cells were treated with actinomycin D (an inhibitor of transcription). qRT-PCR assays showed that the circFLNA transcript with a half-life of more than 24 h was more stable than linear FLNA mRNA in HIEC-6 cells (Fig. 2B). In addition, total RNAs extracted from HIEC-6 cells were digested with RNase R prior to qRT-PCR. We noted that circFLNA was more resistant to RNase R than linear FLNA mRNA (Fig. 2C). RNAs extracted from nuclear and cytoplasmic samples was detected by RT-PCR. The results showed that circFLNA was mainly localized in the cytoplasm of HIEC-6 cells, rather than in the nucleus (Fig. 2D). Moreover, FISH assays showed that circFLNA was predominantly expressed in the cytoplasm of LPS-treated HIEC-6 cells compared to controls (Fig. 2E). Taken together, these results indicate that circFLNA was upregulated in LPS-treated HIEC-6 cells and was mainly localized in the cytoplasm.

### circFLNA inhibits cell viability and promotes apoptosis and inflammation of LPS-treated HIEC-6 cells

**Fig. 3:**
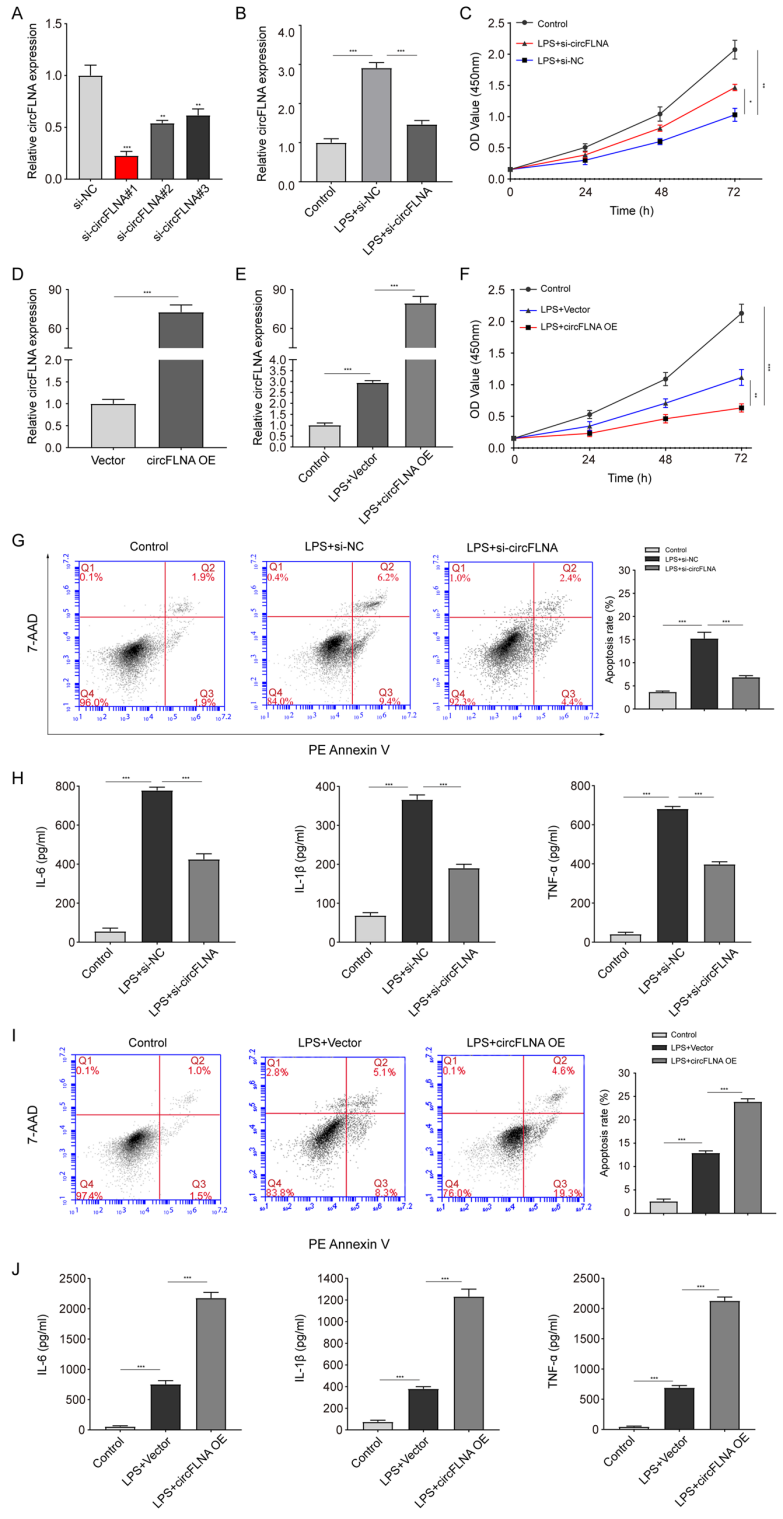
circFLNA inhibits cell proliferation, as well as promotes apoptosis and inflammation of LPS-treated HIEC-6 cells. **A** The reduced efficiency of three siRNAs targeting circFLNA was verified by qRT-PCR in HIEC-6 cells. **B** qRT-PCR assays were used to detect circFLNA expression in LPS-treated HIEC-6 cells transfected with si-circFLNA#1 or the negative control (si-NC). **C** Cell Counting Kit-8 assays were used to evaluate the proliferation of LPS-treated HIEC-6 cells transfected with si-circFLNA#1 (red line) or si-NC (blue line). **D** circFLNA expression was detected by qRT-PCR in HIEC-6 cells infected with lentivirus overexpressing circFLNA or with the empty control vector. **E** qRT-PCR assays were used to detect circFLNA expression in LPS-treated HIEC-6 cells transfected with circFLNA OE or vector. **F** Cell Counting Kit-8 assays were used to evaluate the proliferation of LPS-treated HIEC-6 cells transfected with circFLNA OE (red line) or vector (blue line). **G** Cell apoptosis was evaluated using flow cytometry in LPS-treated HIEC-6 cells transfected with si-circFLNA#1 or si-NC. **H** Concentrations of IL-6, IL-1β, and TNF-α in the supernatants of LPS-treated HIEC-6 cells transfected with si-circFLNA#1 or si-NC were measured by ELISA. **I** Apoptosis was evaluated by flow cytometry in LPS-treated HIEC-6 cells transfected with circFLNA OE or vector. **J** Concentrations of IL-6, IL-1β, and TNF-α in supernatants of LPS-treated HIEC-6 cells transfected with circFLNA OE or vector were measured using ELISA. Values are shown as mean ± standard deviation *P < 0.05, **P < 0.01, and ***P < 0.001

To evaluate the biological role of circFLNA in sepsis, interference and overexpression assays were utilized in this study. First, three siRNAs targeting the junction sites of circFLNA were designed, and si-circFLNA#1 displayed the highest interference efficiency (Fig. 3A). The interference efficiency of si-circFLNA#1 was verified in LPS-treated HIEC-6 cells (Fig. 3B). In addition, we found that decreased expression of circFLNA blocked LPS-induced inhibition of cell viability (Fig. 3C). Upregulation of circFLNA was observed after transfection with lentivirus circFLNA OE in HIEC-6 cells (Fig. 3D and E). Accordingly, increased expression of circFLNA aggravated LPS-induced inhibition of cell viability (Fig. 3F). Furthermore, we found that decreased circFLNA expression ameliorated apoptosis (Fig. 3G) and inflammation factor levels (IL-6, IL-1β, and TNF-α) in LPS-treated HIEC-6 cells (Fig. 3H). In contrast, circFLNA OE aggravated apoptosis (Fig. 3I) and inflammation factor levels (IL-6, IL-1β, and TNF-α) in LPS-treated HIEC-6 cells (Fig. 3J). Taken together, these results indicate that circFLNA inhibited cell viability and promoted apoptosis and inflammation in LPS-treated HIEC-6 cells.

### circFLNA promotes apoptosis and inflammation by regulating Fas in LPS-treated HIEC-6 cells

**Fig. 4:**
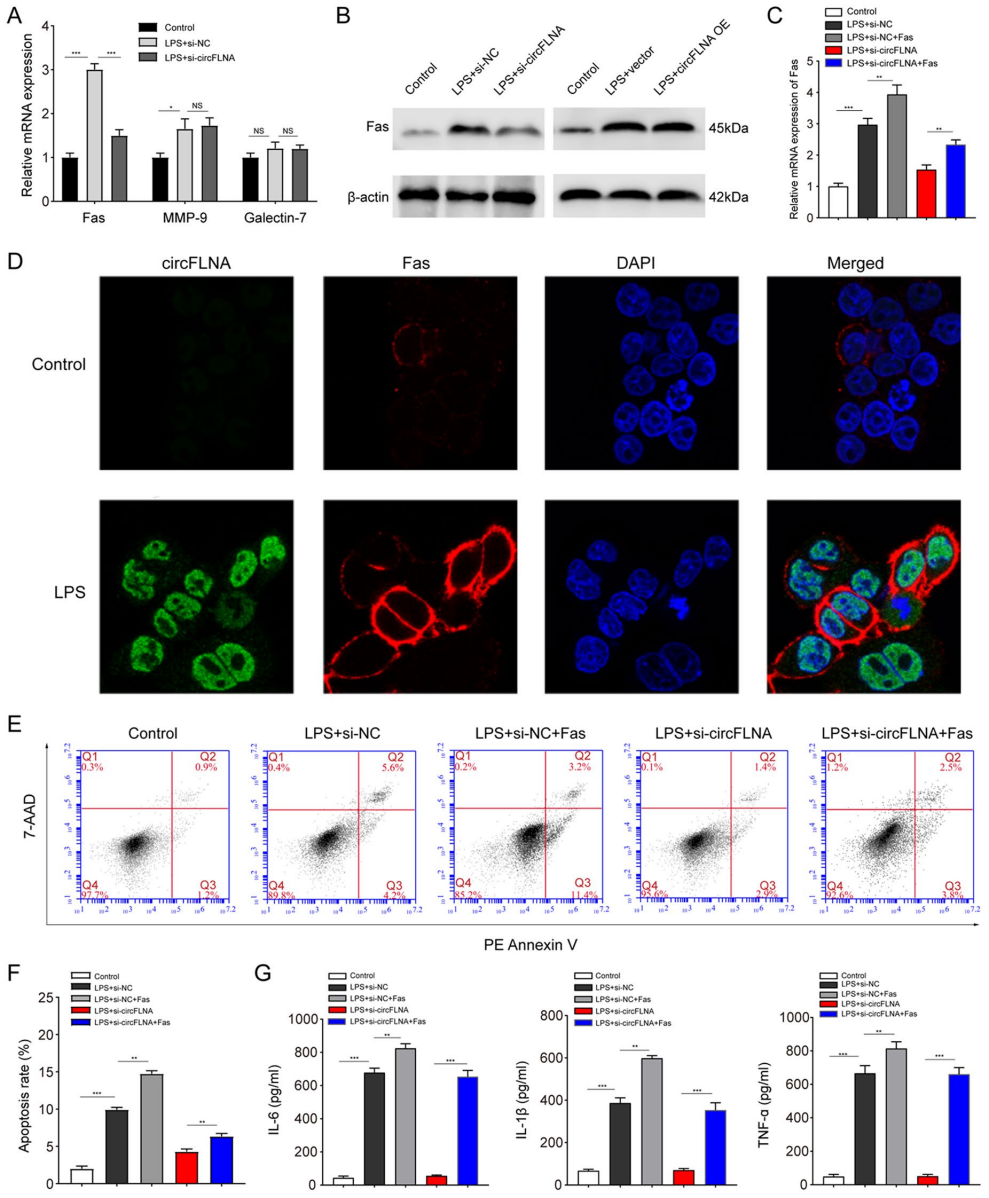
circFLNA promotes apoptosis and inflammation by regulating Fas in LPS-treated HIEC-6 cells. **A** qRT-PCR assays were performed to detect the expression of the three predicted genes in LPS-treated HIEC-6 cells transfected with si-circFLNA#1 or si-NC. **B** Western blot assays evaluated the protein levels of Fas in LPS-treated HIEC-6 cells transfected with si-circFLNA#1, si-NC, circFLNA OE, or vector. **C** Fas mRNA expression was detected in LPS-treated HIEC-6 cells that were co-transfected with lentivirus expressing si-circFLNA#1 or Fas. **D** FISH and immunofluorescence assays were performed separately to detect the expression of circFLNA and Fas in LPS-treated HIEC-6 cells. Note: Green (circFLNA), blue (DAPI, which reflects total cells), and red (Fas). **E** and **F** Cell apoptosis was evaluated by flow cytometry in LPS-treated HIEC-6 cells that were co-transfected with adenovirus expressing si-circFLNA#1 or Fas.** G** Concentrations of IL-6, IL-1β, and TNF-α in supernatants of LPS-treated HIEC-6 cells that were co-transfected with adenovirus expressing si-circFLNA#1 or Fas were measured by ELISA. Values are shown as mean ± standard deviation. *NS* not significant. *P < 0.05, **P < 0.01, and ***P < 0.001

To determine the mechanisms of circFLNA in promoting HIEC-6 cell apoptosis and inflammation, protein array analysis was performed in LPS-treated HIEC-6 cells with increased circFLNA expression. The results showed that levels of five proteins were decreased (FC < 0.3, *P* < 0.05), while those of three proteins were increased (FC > 3, *P* < 0.05) in LPS-treated HIEC-6 cells with increased circFLNA expression (Table 2). qRT-PCR assays were performed to verify the mRNA expression of the three upregulated proteins. We found that decreased expression of circFLNA ameliorated the upregulation of Fas (Fas cell surface death receptor) induced by LPS but did not influence the expression of MMP-9 (matrix metalloproteinase-9) and TIM-1 (T-cell immunoglobulin mucin-1) (Fig. 4A). Furthermore, western blot assays confirmed that the protein level of Fas was reduced after transfection with si-circFLNA#1 in LPS-treated HIEC-6 cells, whereas it was elevated in cells transfected with circFLNA OE (Fig. 4B). Fas, also known as CD95, is constitutively expressed on the basolateral surface of IECs, mediates apoptosis after activation by Fas ligand (FasL), and promotes the production of cytokines and chemokines following LPS treatment [34, 35]. Therefore, we aimed to determine whether circFLNA promotes apoptosis and inflammation by increasing Fas expression in LPS-treated HIEC-6 cells. As shown in Fig. 4C, interference of circFLNA by si-circFLNA#1 significantly decreased the mRNA expression of Fas, which was restored after transfection with lentivirus overexpressing Fas in LPS-treated HIEC-6 cells. Moreover, FISH assays demonstrated that circFLNA and Fas were located in the cytoplasm and cytomembrane, respectively, and that both were upregulated in LPS-treated HIEC-6 cells (Fig. 4D). Finally, we found that the restoration of Fas expression significantly abrogated the effect of si-circFLNA on apoptosis (Fig. 4E and F) and inflammation factor levels (IL-6, IL-1β, and TNF-α) in LPS-treated HIEC-6 cells (Fig. 4G). Taken together, these results indicate that circFLNA promoted apoptosis and inflammation by regulating Fas in LPS-treated HIEC-6 cells.

**Table 2:** Differentially expressed proteins in LPS-treated Caco2 cells compared with controls

Proteins	Fold change
Fas cell surface death receptor (Fas)	4.31
Matrix metalloproteinase-9 (MMP-9)	4.02
Galectin-7	3.56
T-cell immunoglobulin and mucin domain-containing protein-2 (TIM-2)	0.29
Oncostatin M (OSM)	0.27
Vascular cellular adhesion molecule-1 (VCAM-1)	0.25
Insulin-like growth factor binding protein-4 (IGFBP-4)	0.17
Epithelial cadherin (E-cadherin)	0.13

### circFLNA acts as a miRNA sponge of miR-766-3p in sepsis

**Fig. 5:**
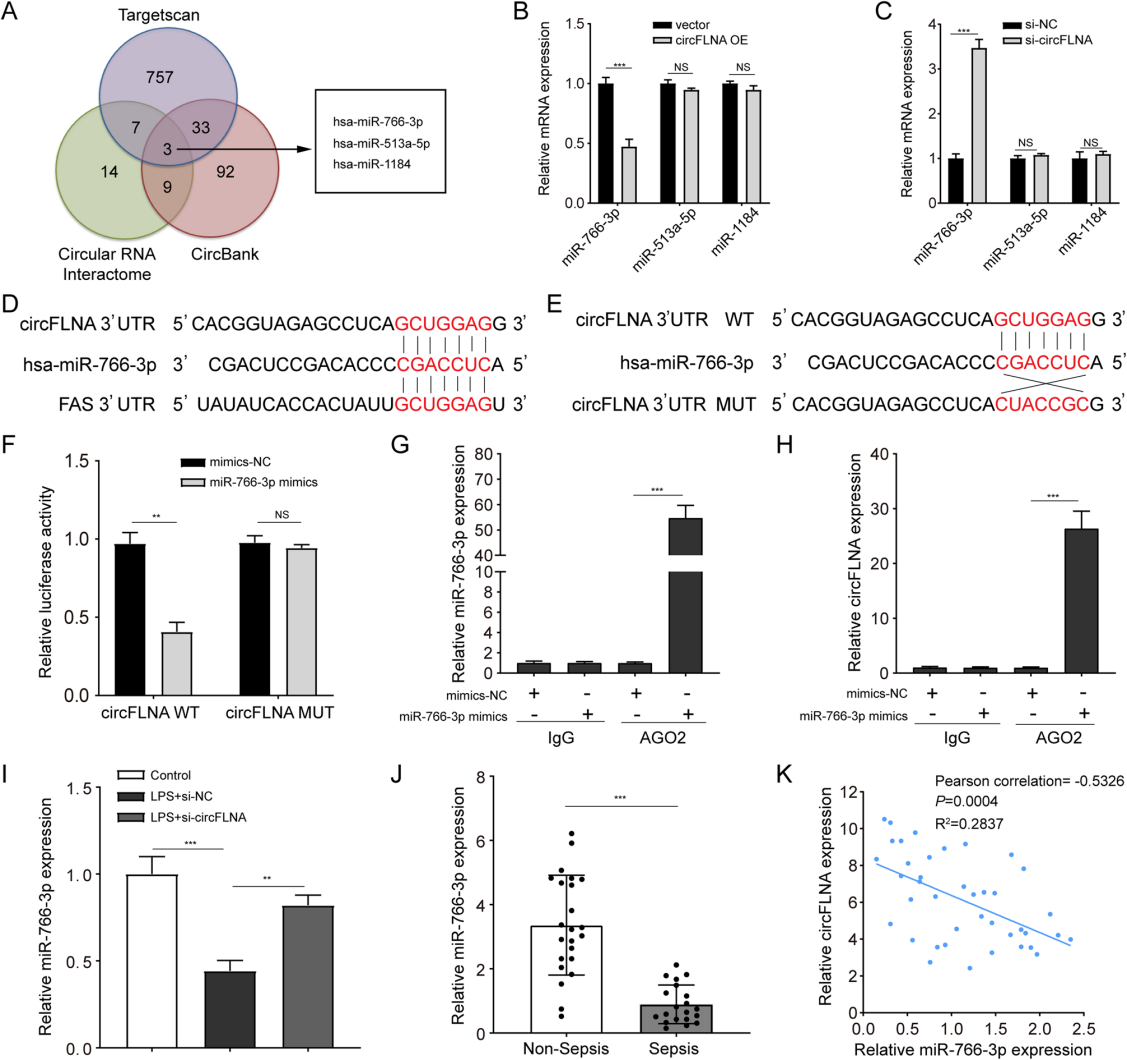
circFLNA act as a miRNA sponge of miR-766-3p in sepsis. **A** Schematic illustration showing the identification of three miRNAs, hsa-miR-766-3p, hsa-miR-513a-5p, and hsa-miR-1184, as predicted by circBank, circInteractome, and TargetScan. **B** and **C** qRT-PCR assays were performed to detect the expression of three miRNAs in circFLNA-overexpressing and -silenced HIEC-6 cells. **D** The putative binding sites of miR-766-3p on circFLNA and Fas were identified by bioinformatic analysis. **E** Predicted binding sites between miR-766-3p and wild-type (WT) or mutant (MUT) circFLNA sequences. **F** Dual-luciferase reporter assays were performed in HIEC-6 cells co-transfected with WT circFLNA or MUT circFLNA and miR-766-3p mimics or NC. **G** and **H** The expression of miR-766-3p and circFLNA was detected by qRT-PCR after the RIP assay for AGO2 in HIEC-6 cells transfected with miR-766-3p mimics or NC. **I** qRT-PCR assays were used to detect the expression of miR-766-3p in LPS-treated HIEC-6 cells transfected with si-circFLNA#1 or NC. **J** Expression of miR-766-3p in the intestinal mucosa of 20 patients with sepsis and 23 non-sepsis patients was detected by qRT-PCR. **K** The circFLNA expression and miR-766-3p were negatively correlated in the intestinal mucosa from 40 patients with sepsis (Pearson correlation: − 0.5326, *P* = 0.0004, *R*^2^ = 0.2837)

CircRNAs have been reported to act as miRNA sponges in the cytoplasm [36]. As mentioned above, circFLNA was mainly localized in the cytoplasm and was stably expressed in LPS-treated HIEC-6 cells. Therefore, we investigated whether circFLNA promoted apoptosis and inflammation in LPS-treated HIEC-6 cells by binding to certain miRNAs. We then performed bioinformatic analysis using three databases: CircInteractome (https://circinteractome.nia.nih.gov/index.html), circBank (http://www.circbank.cn/), and TargetScan (https://www.targetscan.org/vert_80/) were used to predict miRNAs. We identified three overlapping miRNAs (hsa-miR-766-3p, hsa-miR-513a-5p, and hsa-miR-1184) that potentially bind to circFLNA and the 3′-untranslated region (UTR) of Fas mRNA (Fig. 5A). The miRNAs predicted by CircInteractome, CircBank, and TargetScan are listed in Supplementary Table S2. As a result, we speculated that circFLNA might sponge miRNAs and promote Fas expression in HIEC-6 cells. qRT-PCR was performed to verify the influence of circFLNA on the expression of hsa-miR-766-3p, hsa-miR-513a-5p, and hsa-miR-1184 in HIEC-6 cells. The results showed that miR-766-3p expression was significantly decreased in HIEC-6 cells transfected with circFLNA OE (Fig. 5B), while it significantly increased with interference of circFLNA (Fig. 5C). In addition, changes in circFLNA expression had no effect on hsa-miR-513a-5p or hsa-miR-1184. The putative binding sites of miR-766-3p on circFLNA and Fas are shown in Fig. 5D.

Furthermore, we performed dual-luciferase assays to determine whether circFLNA directly binds to miR-766-3p. Luciferase reporter plasmids containing the complementary seed sequence of circFLNA at the 3′-UTR of circFLNA were constructed (Fig. 5E). The results showed that co-transfection with WT circFLNA vector and miR-766-3p mimics significantly reduced luciferase activity, but not when MUT circFLNA vector was transfected in HIEC-6 cells (Fig. 5F). Previous work has demonstrated that miRNAs regulate mRNA translation in an Argonaute 2 (AGO2)-dependent manner [37]. Therefore, a RIP assay for AGO2 in HIEC-6 cells was performed to confirm the direct binding between miR-766-3p and circFLNA. Relative to IgG immunoprecipitation, circFLNA and miR-766-3p were both enriched in AGO2 immunoprecipitation, indicating that circFLNA is involved in miR-766-3p-mediated mRNA translation (Fig. 5G and H). Additionally, we found that the expression of miR-766-3p decreased following treatment with LPS and could be restored by circFLNA interference (Fig. 5I). We detected the expression of miR-766-3p in the intestinal mucosae obtained from 20 patients with sepsis and 23 non-sepsis patients by qRT-PCR. The results showed that miR-766-3p expression was significantly reduced in patients with sepsis compared with that in non-septic patients (Fig. 5J). We also found that miR-766-3p was negatively correlated with circFLNA in the intestinal mucosae of patients with sepsis (n = 40, Pearson correlation: -0.5326, *P* = 0.0004, *R*^2^ = 0.2837) (Fig. 5K). In conclusion, circFLNA acts as a miRNA sponge for miR-766-3p in sepsis.

### miR-766-3p inhibits apoptosis and inflammation in LPS-treated HIEC-6 cells by directly targeting Fas

**Fig. 6:**
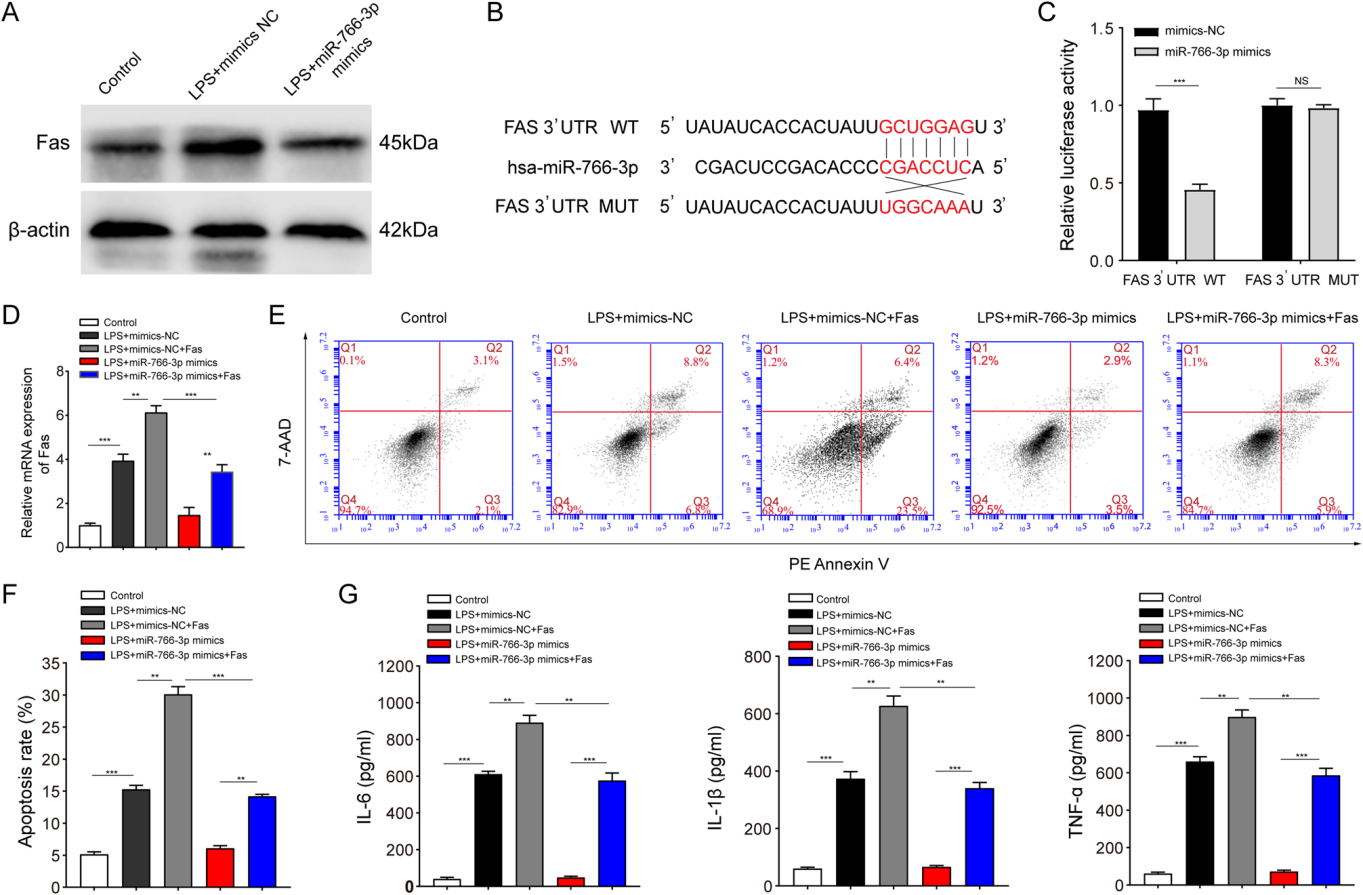
miR-766-3p inhibits apoptosis and inflammation by directly targeting Fas in LPS-treated HIEC-6 cells. **A** The expression of Fas was detected by western blotting in LPS-treated HIEC-6 cells transfected with miR-766-3p mimics or NC. **B** Schematic diagram showing that the 3′-UTR of Fas mRNA contains a complementary site for the seed region of miR-766-3p. The mutant sequence was used as a negative control for the luciferase reporter assay. **C** Dual-luciferase reporter assays were performed to detect the influence of miR-766-3p mimics on the luciferase activities of Fas 3′-UTR WT and MUT reporter genes. **D** qRT-PCR assays were performed to detect the expression of Fas in LPS-treated HIEC-6 cells that were co-transfected with adenovirus expressing miR-766-3p mimics or Fas. **E** and **F** Cell apoptosis was evaluated by flow cytometry in LPS-treated HIEC-6 cells that were co-transfected with adenovirus expressing miR-766-3p mimics or Fas. **G** Concentrations of IL-6, IL-1β, and TNF-α in supernatants of LPS-treated HIEC-6 cells that were co-transfected with adenovirus expressing miR-766-3p mimics or Fas were measured by ELISA. Values are shown as mean ± standard deviation. *NS* not significant. *P < 0.05, **P < 0.01, and ***P < 0.001

As mentioned above, Fas is a potential target gene of miR-766-3p. Western blot assays showed that overexpression of miR-766-3p prevented LPS-induced upregulation of Fas (Fig. 6A). To confirm whether Fas was directly targeted by miR-766-3p, the 3′-UTR of Fas mRNA containing a complementary binding site to miR-766-3p was cloned into a luciferase reporter plasmid (Fig. 6B). The luciferase reporter assay showed that co-transfection with miR-766-3p mimics significantly decreased the luciferase activity of the WT Fas 3′-UTR reporter but not the MUT Fas 3'-UTR reporter in HIEC-6 cells (Fig. 6C). These results indicate that Fas is a direct target of miR-766-3p. As shown in Fig. 6D, miR-766-3p mimics significantly reduced the mRNA expression of Fas, which was restored after transfection with lentivirus overexpressing Fas in LPS-treated HIEC-6 cells. Moreover, we found that restoration of Fas expression significantly deteriorated the effect of miR-766-3p mimics on apoptosis (Fig. 6E and F) and inflammation factor levels (IL-6, IL-1β, and TNF-ɑ) in LPS-treated HIEC-6 cells (Fig. 6G). Taken together, these findings indicate that miR-766-3p inhibits apoptosis and inflammation in LPS-treated HIEC-6 cells by directly targeting Fas.

### CircFLNA promotes Fas-associated apoptosis by targeting miR-766-3p

**Fig. 7:**
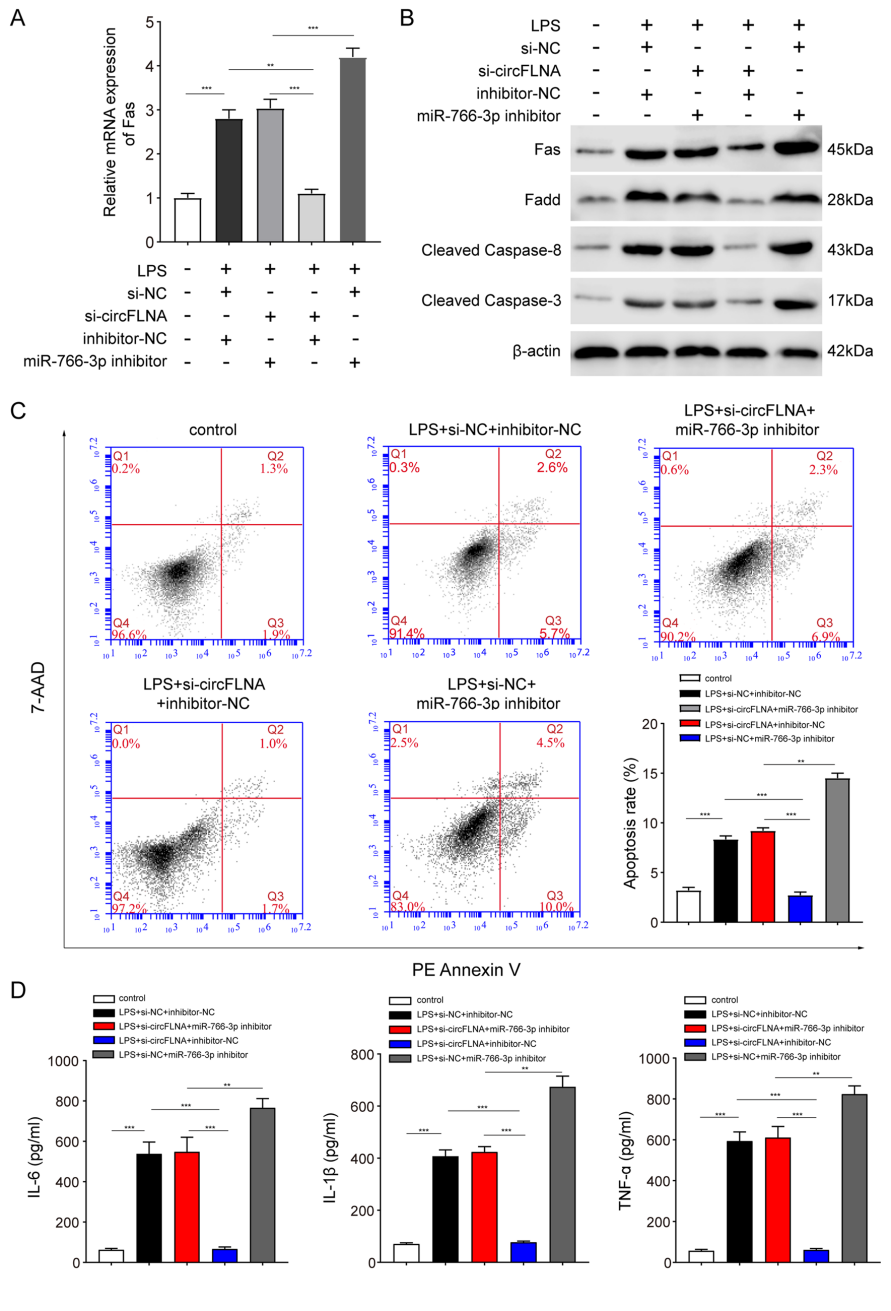
circFLNA promotes Fas-associated apoptosis by targeting miR-766-3p.** A** and **B** Downregulation of Fas mRNA **(A)** and apoptosis-associated proteins **(B)** in LPS-treated HIEC-6 cells transfected with si-circFLNA#1 was partially reversed by inhibition of miR-766-3p. **C** Apoptosis was evaluated by flow cytometry in LPS-treated HIEC-6 cells that were co-transfected with adenovirus expressing si-circFLNA#1 or miR-766-3p inhibitor. Histograms represent the proportion of apoptotic cells. **D** Concentrations of IL-6, IL-1β, and TNF-α in supernatants of LPS-treated HIEC-6 cells that were co-transfected with adenovirus expressing si-circFLNA#1 or miR-766-3p inhibitor were measured by ELISA. Values are shown as mean ± standard deviation. *P < 0.05, **P < 0.01, and ***P < 0.001

Given that both circFLNA and miR-766-3p target Fas in the apoptosis and inflammation of LPS-treated HIEC-6 cells (Figs. 4 and 6), we hypothesized that circFLNA might exert its biological effect on Fas by sponging miR-766-3p. Both qRT-PCR and western blot assays showed that co-transfection with the miR-766-3p inhibitor partially abrogated the downregulation of Fas induced by circFLNA interference in LPS-treated HIEC-6 cells (Fig. 7A and B). The Fas-associated apoptosis pathway begins with FasL binding to Fas, and then the adaptor protein, Fas-associated death domain protein (FADD), recruits and activates caspase-8. Consequently, caspase-3 is activated, which contributes to the activation of the protease cascade, leading to apoptosis [38, 39]. In this study, we found that the protein levels of FADD, cleaved caspase-8, and cleaved caspase-3 were decreased after interference with circFLNA, and this effect was blocked by miR-766-3p inhibitor (Fig. 7B). Subsequently, we demonstrated that inhibition of miR-766-3p impaired the si-circFLNA-mediated inhibition of apoptosis and inflammation factor levels (IL-6, IL-1β, and TNF-α) in LPS-treated HIEC-6 cells (Fig. 7C and D). Collectively, these results reveal that circFLNA enhanced Fas expression by targeting miR-766-3p in LPS-treated HIEC-6 cells, followed by increased Fas-associated apoptosis.

### CircFLNA interference ameliorates intestinal epithelial injury and inflammation by regulating miR-766-3p in vivo

**Fig. 8:**
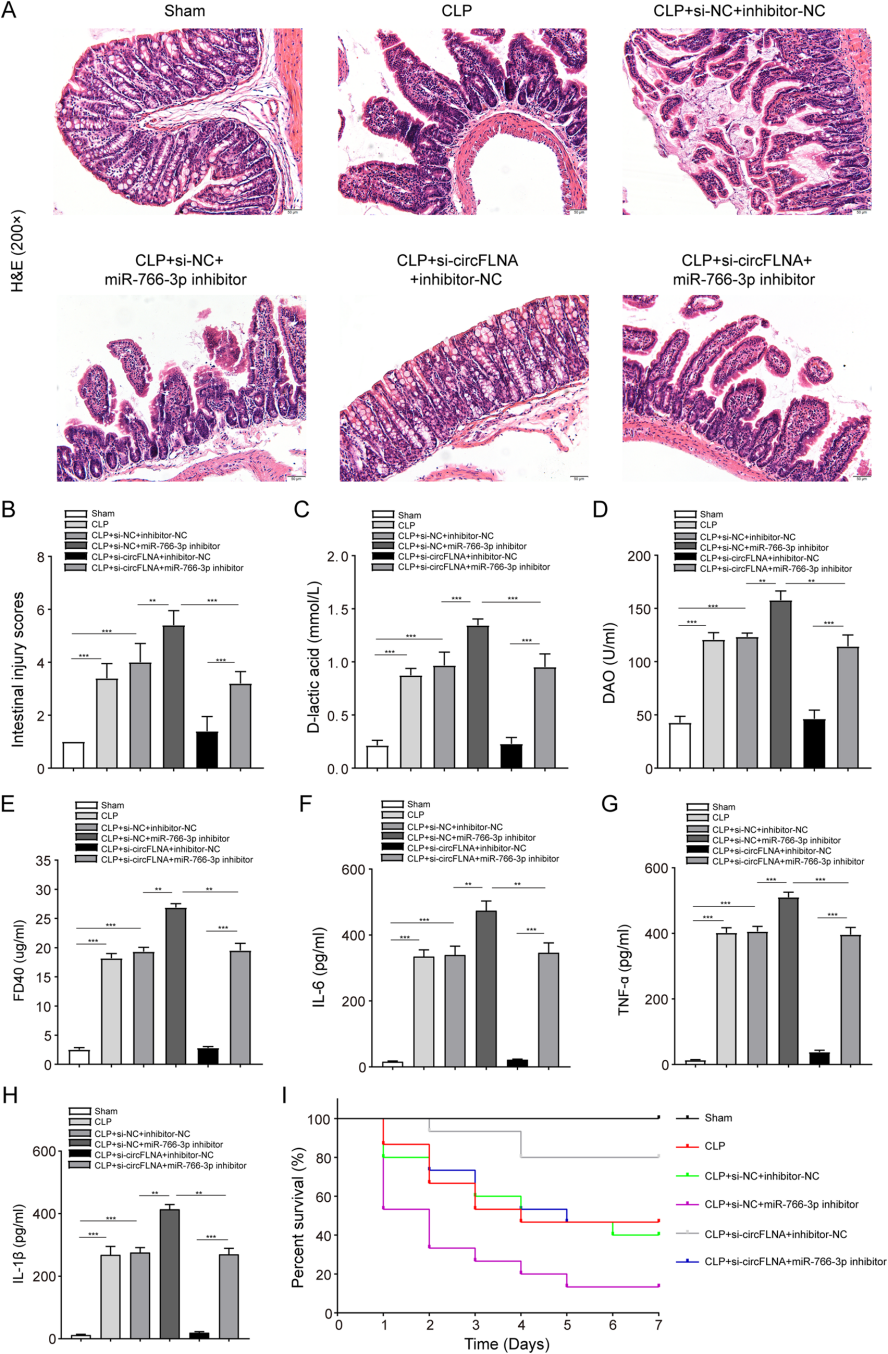
Inhibition of circFLNA ameliorates intestinal epithelial injury and inflammation by regulating miR-766-3p in vivo. **A** Representative images of H&E staining indicate that injection of miR-766-3p inhibitor impaired the improvement effect of si-circFLNA on intestinal injury at 48 h after CLP (H&E staining 200 ×). **B** Histological injury of the intestines from each group was assessed using Chiu’s intestinal injury score. **C, D**, and **E** Levels of D-lactic acid, DAO, and FD40 in the serum of mice from each group. **F, G**, and **H** Levels of inflammatory factors, such as IL-6, TNF-α, and IL-1β, in the intestinal mucosa of mice were measured by ELISA. Kaplan–Meier survival curves and log-rank tests were used to analyze the survival rates of mice from each group. Values are shown as mean ± standard deviation. *P < 0.05, **P < 0.01, and ***P < 0.001

To elucidate the roles of circFLNA and miR-766-3p in intestinal epithelial injury during sepsis in vivo, a CLP model was constructed with C57BL/6 mice. H&E staining was utilized to detect histopathological changes in the intestinal epithelium, and representative images of each group are shown in Fig. 8A. Chiu’s intestinal injury score was used to quantify the degree of histological injury (Fig. 8B). The images showed that the injection of si-circFLNA ameliorated intestinal injury, and the CLP + si-circFLNA + inhibitor-NC group exhibited a intestinal epithelial structure similar to that of the sham group. Both groups did not show tissue edema, mucosal atrophy and necrosis, villous rupture, or mucus thinning, which appeared in the other four groups. Compared to the CLP + si-circFLNA + inhibitor-NC group, the CLP + si-circFLNA + miR-766-3p inhibitor group exhibited severe intestinal injury. The results showed that the injection of the miR-766-3p inhibitor impaired the effect of si-circFLNA on intestinal injury. Accordingly, the CLP + si-NC + miR-766-3p inhibitor group exhibited more obvious mucosal necrosis, shedding, and villous rupture than the other groups did.

Furthermore, to study the impact of circFLNA and miR-766-3p on intestinal mucosal permeability, the levels of D-lactic acid, DAO, and FD-40 were detected in serum samples obtained from mice 48 h after CLP. The levels of D-lactic acid, DAO, and FD-40 in mouse serum were significantly downregulated after injection of si-circFLNA, whereas injection of miR-766-3p inhibitor impaired the improvement effect of si-circFLNA on intestinal mucosal permeability (Fig. 8C, D, and E). To determine the impact of circFLNA and miR-766-3p on inflammation of the intestinal mucosa, the levels of TNF-α, IL-6, and IL-1β were measured 48 h after CLP. The levels of TNF-α, IL-6, and IL-1β in the intestinal mucosa of mice were significantly decreased after injection of si-circFLNA, whereas injection of miR-766-3p inhibitor blocked the effect of si-circFLNA on intestinal inflammation (Fig. 8F, G, and H). In addition, the Kaplan–Meier survival curves of mice in each group for 0–7 days post CLP surgery were plotted (Fig. 8I). The mice in the sham group survived for 7 days. The survival rate was not statistically different between the sham group and the CLP + si-circFLNA + inhibitor-NC group pre-treated with si-circFLNA (100% vs. 80%, *P* = 0.073). Compared to the CLP + si-circFLNA + inhibitor-NC group, the CLP + si-circFLNA + miR-766-3p inhibitor group exhibited a lower survival rate (80% vs. 46.7%, *P* = 0.047). The lowest survival rate was observed in CLP + si-NC + miR-766-3p inhibitor group. The results showed that pre-treatment with the miR-766-3p inhibitor reduced the effect of si-circFLNA on survival. These findings suggest that interference with circFLNA ameliorates intestinal epithelial injury and inflammation by regulating miR-766-3p in vivo.

### CircFLNA promotes Fas-associated apoptosis by targeting miR-766-3p in vivo

**Fig. 9:**
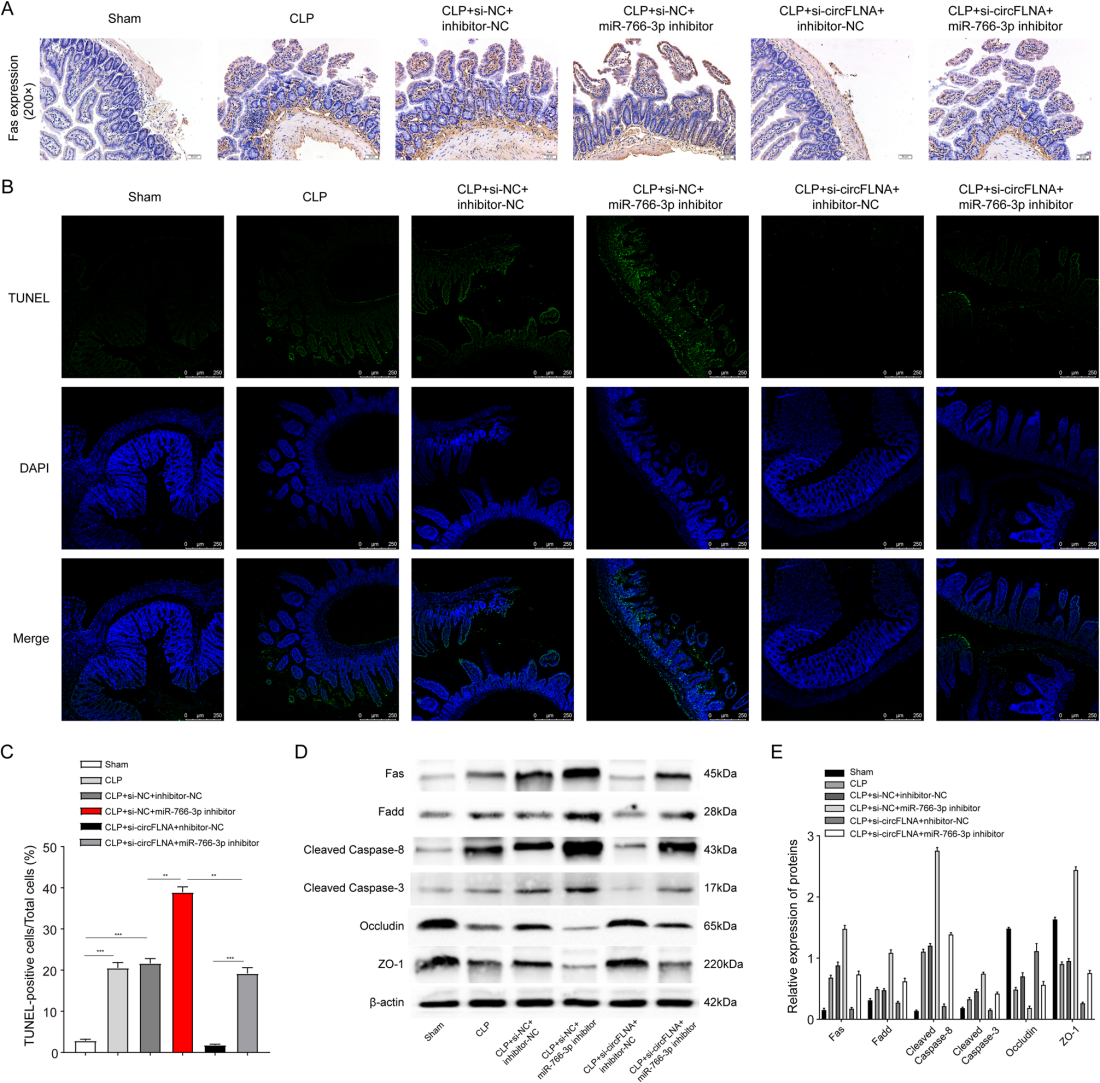
CircFLNA promotes Fas-associated apoptosis by targeting miR-766-3p in vivo. **A** The expression of Fas in the mouse intestinal epithelium was detected by immunohistochemical staining. **B** and **C** TUNEL assay was performed to detect apoptosis in the mouse intestinal epithelium. Note: Green (FITC that reflects apoptotic cells); blue (DAPI that reflects total cells). Scale bars: 250 µm. **D** and **E** The levels of occludin, ZO-1, and Fas-associated apoptotic proteins in mouse intestinal epithelium were detected by western blot analysis. Values are shown as mean ± standard deviation. *P < 0.05, **P < 0.01, and ***P < 0.001

To elucidate whether circFLNA and miR-766-3p exert their biological effects depending on Fas in the CLP model, we performed IHC staining to detect Fas expression in the intestinal epithelium. Representative images from each group are shown in Fig. 9A. CLP upregulated the number of Fas-positive cells in the intestinal epithelium. The number of Fas-positive cells was not different between the sham group and the CLP + si-circFLNA + inhibitor-NC group pre-treated with si-circFLNA. The number of Fas-positive cells was higher in the CLP + si-circFLNA + inhibitor-NC group than in the CLP + si-circFLNA + miR-766-3p inhibitor group. The highest expression of Fas was observed in the CLP + si-NC + miR-766-3p inhibitor group. These results show that interference with circFLNA could reduce the expression of Fas, which could be restored by the miR-766-3p inhibitor. It has been reported that Fas-induced apoptosis is involved in epithelial cell loss in the gut [40]. This led us to perform a TUNEL assay to detect the apoptosis rate of the intestinal epithelium (Fig. 9B and C). The images show that the apoptosis rate of the intestinal epithelium was in line with the Fas level. The execution of CLP increased the apoptosis rate of the intestinal epithelium, which could be restored by interference with circFLNA, but worsened when pre-treated with miR-766-3p inhibitor. In addition, western blotting was performed to detect the proteins involved in the Fas/FasL signaling pathway. The expression of Fas, FADD, cleaved caspase 8, cleaved caspase 3, occludin, and ZO-1 proteins are shown in Fig. 9D and E. We found that the protein levels of Fas, FADD, cleaved caspase-8, and cleaved caspase-3 in the intestinal mucosa were decreased after interference with circFLNA, and this effect was blocked by miR-766-3p inhibitor. As important components of the tight junction of the intestinal epithelium, the expression of occludin and ZO-1 is reduced when apoptosis occurs in the intestinal epithelium [41]. In this study, the protein levels of occludin and ZO-1 in the intestinal mucosa were increased after interference with circFLNA and decreased after injection of the miR-766-3p inhibitor. These results indicate that circFLNA promotes Fas-associated apoptosis by targeting miR-766-3p in vivo.

## Discussion

**Fig. 10:**
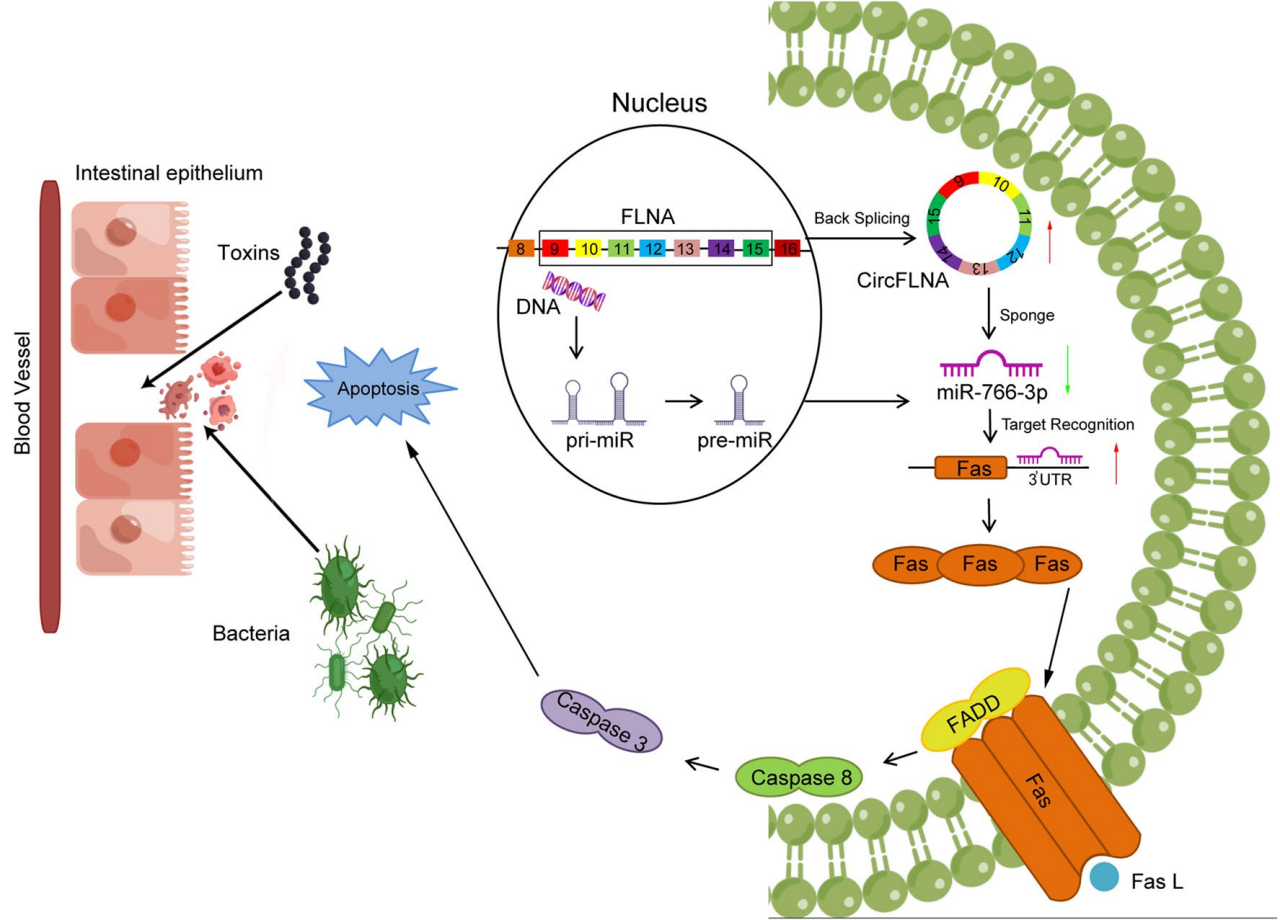
Schematic diagram showing that circFLNA promotes intestinal epithelial apoptosis and inflammation through the miR-766-3p/Fas axis. circFLNA acts as a competing endogenous RNA to regulate miR-766-3p, resulting in the enhancement of Fas expression, thereby attenuating intestinal barrier function by promoting intestinal epithelial apoptosis and inflammatory response

Intestinal epithelial integrity prevents harmful substances, such as bacteria and toxins, from passing through the intestinal mucosa and entering the blood circulation. Increased apoptosis of intestinal epithelial cells induced by sepsis exacerbates the progression of sepsis [33]. Therefore, exploring the molecular mechanisms of intestinal epithelial apoptosis and identifying promising targets will contribute to the prevention and treatment of abdominal sepsis. In this study, we obtained purified epithelial cells from intestinal crypts using LCM and found that circFLNA was upregulated in the intestinal epithelium after intestinal perforation-induced abdominal sepsis. CircFLNA promoted apoptosis and inflammation of intestinal epithelial cells in both cultured HIEC-6 cells and CLP mouse models. The molecular mechanism analysis of circFLNA revealed that it enhanced the Fas-mediated apoptosis signaling pathway by sponging miR-766-3p. These findings indicated that the circFLNA/miR-766-3p/Fas axis plays a significant role in the pathogenesis of intestinal epithelial injury (Fig. 10).

Diverse subtypes of IECs present in the intestinal epithelium consist of intestinal stem, Paneth, goblet, enteroendocrine, tuft, and microfold cells, and absorptive enterocytes, and are distributed along the crypt–villus axis [42]. In contrast to other cells, ISCs and Paneth cells are located in the intestinal crypts rather than in the villi. ISCs contribute to the constant renewal of the intestinal epithelium and regulate intestinal homeostasis by maintaining stemness [43]. In addition, Paneth cells can resist the invasion of potential pathogens by self-generated antibiotics and participate in the innate antimicrobial response [44]. Despite the vital role of intestinal crypts, few studies have elucidated the ectopic expression of circRNAs in subtype cells of intestinal crypts in abdominal sepsis. In this study, we performed LCM in four patients with intestinal perforation-induced abdominal sepsis and non-sepsis patients to obtain the specific cell types of the crypt epithelium from the entire intestinal mucosa. CircFLNA was found to be upregulated in the intestinal epithelium exposed to abdominal sepsis using circRNA microarray and was validated in patients with intestinal perforation and CLP mouse models.

Many studies have revealed that circRNAs can function as sponges, interact with RNA-binding proteins (RBPs), and regulate gene translation [10]. As the primary function, circRNA decreases the expression of miRNA by sponging it and thereby increases the translation of mRNA, which is targeted by miRNA [11]. circRNAs have been shown to be involved in inflammation, immunosuppression, coagulation dysfunction, and organ dysfunction during sepsis [45]. In this study, we found that circFLNA was upregulated in LPS-treated HIEC-6 cells and promoted apoptosis and inflammation by increasing the expression of the apoptosis-related Fas gene. Bioinformatic analyses showed that circFLNA and Fas bind to miR-766-3p at the same sequence. Consequently, we speculated that circFLNA promoted apoptosis and inflammation of HIEC-6 cells through the miR-766-3p/Fas axis. Several assays, including RNA-FISH, RIP, and dual-luciferase reporter assays, demonstrated that the majority of circRNAs were distributed in the cytoplasm and bound with miR-766-3p as an miRNA sponge. qRT-PCR assay showed that miR-766-3p was downregulated in HIEC-6 cells treated with LPS and decreased in the intestinal epithelium of individuals with abdominal sepsis compared with that in non-sepsis samples. miR-766-3p suppresses inflammation in human rheumatoid arthritis and attenuates oxidative injury of chondrocytes but has not been studied in sepsis [46, 47]. The functional assay showed that miR-766-3p could reverse the pro-apoptotic and pro-inflammatory roles of Fas. In addition, we found that circFLNA interference decreased the apoptosis rate and inflammation induced by the miR-766-3p inhibitor in LPS-treated HIEC-6 cells. Taken together, we demonstrated that circFLNA functions as a sponge of miR-766-3p to promote apoptosis and inflammation by upregulating Fas expression in vivo.

The integrity of the intestinal epithelium is critical for defense against environmental and microbial attacks from the gut. Apoptosis of the intestinal epithelium in both patients with abdominal sepsis and mouse CLP models contributed to increased permeability of the intestine and translocation of bacteria from the enteric cavity to the blood [33]. Fas is a member of the TNF receptor family and participates in the extrinsic pathway of apoptosis upon activation by FasL [48]. In the mouse CLP model, circFLNA aggravated intestinal injury and inflammatory response through the Fas-mediated apoptosis pathway by sponging miR-766-3p.

To the best of our knowledge, this is the first study to demonstrate that circFLNA is upregulated in intestinal epithelium during abdominal sepsis using a circRNA microarray. Furthermore, we demonstrated that circFLNA aggravated apoptosis and the inflammatory response through the Fas-mediated apoptosis pathway by sponging miR-766-3p in both LPS-treated HIEC-6 cells and a mouse CLP model. However, the current study has some limitations. First, we validated the expression of circFLNA in only 20 patients with abdominal sepsis and 23 non-sepsis patients because it was challenging to obtain suitable samples. This is not sufficient to analyze the relationship between circFLNA and clinical features, as well as the mortality of individuals with abdominal sepsis. As a result, larger sample sizes are required to validate circFLNA expression. In addition, it would be preferable to examine the expression of circFLNA in serum samples from patients with sepsis to facilitate the early diagnosis of intestinal injury.

## Conclusions

In conclusion, our findings indicate that circFLNA interference can reduce injury and inflammation of the intestinal epithelium. The circFLNA/miR-766-3p/Fas axis has potential as a novel therapeutic target for treating intestinal injury in sepsis.

## Supplementary Information

Below is the link to the electronic supplementary material.

Supplementary file1 (DOCX 13 KB)

Supplementary file2 (XLSX 22 KB)

## Data Availability

Data and materials will be shared.
