# Supplementary material for: circFLNA promotes intestinal injury during abdominal sepsis through Fas-mediated apoptosis pathway by sponging miR-766-3p
Source: Inflamm Res. 2023 Jan 10;72(3):509–29. doi: 10.1007/s00011-023-01688-1 (PMC10023616; doi:10.1007/s00011-023-01688-1)
Supplement: Supplementary file 1 — Supplementary file1 (DOCX 13 KB) [file 11_2023_1688_MOESM1_ESM.docx]

**Supplementary Table S1.** Oligonucleotide Sequence for the primers used in the study.

| Name | Oligonucleotide sequence (5' - 3') | |
| --- | --- | --- |
|  | Forward | Reverse |
| circFLNA | CCAGCTGAGGCTCTACCGTGCC | GAGGCGTCAGCATCCCCAACAG |
| circBNC2 | GCAGTTCGGAACCAGAACGAC | ATGCTGGCCAGTCTTGCTCAC |
| circFAM13B | TCTCCCATCAGCATCCTACCA | GCTGTTCCACAGCTGCTCTA |
| circLARP4 | GGGCATCAGGAGCAAACTTA | CTGGCGAATTAAAGCCATTC |
| circEDIL3 | AATGACCAGTCACAATGGTTACAGA | CACTAATGGGTTCCTCGGTTCT |
| MiR-766-3p | ACTCCAGCCCCACAGCC | GAACATGTCTGCGTATCTC |
| MiR-513a-5p | GCGCGTTCACAGGGAGG | AGTGCAGGGTCCGAGGTATT |
| MiR-1184 | GCCGAGCCTGCAGCGACTTG | CTCAACTGGTGTCGTGGA |
| FLNA | ATGCGTCCAAGGTCAAGTG | GTGCCATCAGCGTTGTCT |
| Fas | TGAAGGACATGGCTTAGAAGTG | GGTGCAAGGGTCACAGTGTT |
| MMP-9 | TTGACAGCGACAAGAAGTGG | CCCTCAGTGAAGCGGTACAT |
| TIM-1 | ACATATCGTGGAATCACAACGAC | ACAAGCAGAAGATGGGCATTG |
| GAPDH | AACTTTGGGATTGTGGAAGG | ACACATTGGGGGTAGGAACA |
| U6 | GCTTCGGCAGCACATATACTAAAAT | CGCTTCACGAATTTGCGTGTCAT |
